# Does the Prosperity of a Country Play a Role in COVID-19 Outcomes?

**DOI:** 10.1017/dmp.2020.304

**Published:** 2020-08-12

**Authors:** Amir Khorram-Manesh, Eric Carlström, Attila J. Hertelendy, Krzysztof Goniewicz, Carter B. Casady, Frederick M. Burkle

**Affiliations:** Institute of Clinical Sciences, Sahlgrenska Academy, University of Gothenburg, Gothenburg, Sweden; Department of Research and Development, Swedish Armed Forces Defense Medicine Center, V. Frölunda, Sweden; Institute of Health and Care Sciences, Sahlgrenska Academy, University of Gothenburg, Gothenburg, Sweden; School of Business, University of Southeast Norway, Vestfold, Norway; Department of Information Systems and Business Analytics, College of Business, Florida International University, Miami, FL; Department of Aviation Security, Military University of Aviation, 08-521 Dęblin, Poland; Faculty of the Built Environment, Bartlett School of Construction and Project Management, University College London, United Kingdom; Harvard Humanitarian Initiative, Harvard University and T.H. Chan School of Public Health, Boston, MA

**Keywords:** COVID-19, mortality, pandemic, prosperity, public health emergencies

## Abstract

**Objective:**

This study aims to clarify the association between prosperity and the coronavirus disease (COVID-19) outcomes and its impact on the future management of pandemics.

**Methods:**

This is an observational study using information from 2 online registries. The numbers of infected individuals and deaths and the prosperity rank of each country were obtained from worldometer.info and the Legatum Institute’s Prosperity Index, respectively.

**Results:**

There is a combination of countries with high and low prosperity on the list of COVID-19-infected countries. The risk of the virus pandemic seems to be more extensive in countries with high prosperity. A Spearman’s rho test confirmed a significant correlation between prosperity, the number of COVID-19 cases, and the number of deaths at the 99% level.

**Conclusion:**

New emerging pandemics affect all nations. In order to increase the likelihood of successfully managing future events, it is important to consider preexisting health security, valid population-based management approaches, medical decision-making, communication, continuous assessment, triage, treatment, early and complete physical distancing strategies, and logistics. These elements cannot be taught on-site and on occasion. There is a need for innovative and regular educational activities for all stakeholders committed to safeguarding our future defense systems concerning diagnostic, protection, treatment, and rehabilitation in pandemics, as well as other emergencies.

According to the World Health Organization (WHO), pandemics occur when a new type of virus emerges and spreads around the world. In most cases, pandemics originate from animal influenza viruses, affect all age groups, and result in self-limited illness as well as full recovery without treatment.^1^ These diseases create complex problems because their severity and impact are much higher than seasonal influenza. One reason for this is the fact that a much larger number of people in the population lack preexisting immunity. Additionally, a pandemic can occur in cyclical influenza seasons or defy typical epidemiological patterns, resulting in massive outbreaks during the summer months (eg, H1N1).^1-3^ Coronavirus disease (COVID-19) is no different. It spreads primarily through droplets of saliva or discharge from an infected person´s cough or sneeze. Most people infected with the COVID-19 virus experience mild to moderate respiratory illness; they also recover without requiring special treatment. Vulnerable groups, such as the elderly and those with underlying medical problems, are more likely to develop severe illness. The absence of specific treatments or vaccines for COVID-19 has resulted in different non-pharmacological approaches to prevent and slow down transmission.^4,5^ Countries have reacted differently, with some prioritizing limited restrictions, whereas others have enforced a long period of quarantine, active testing, and symptomatic treatment.^6^

In the past, population-based management (PBM) approaches have been effective in managing pandemics because PBMs assess the unique health conditions and needs of an entire target population. These approaches specifically use efficient and effective measures and/or interventions that are consistent with community-specific cultural, political, and health care values.^2,3^ However, the current approach to the COVID-19 pandemic has largely departed from PBM. The global response to this pandemic has been troublesome and uncoordinated across countries due to lacking emergency public health leadership as well as varying political and economic actions. Consequently, deaths from this pandemic have been significant and policy division between states and countries remains high.^3^

As the number of those infected (and deceased) increase continuously, the situation appears paradoxically worse in more prosperous and developed countries, which are supposed to have sufficient resources and knowledge to protect their population. For instance, 6 out of 10 of the most severely impacted countries listed on worldometer.info at the end of May 2020 were among Europe’s most prosperous.^4,6-10^ According to the Legatum Institute, a London-based independent educational charity institute with a global vision to see all people out of poverty, prosperity consists of the following 12 pillars^11^:

1. *Safety and Security* – the degree to which conflict, terror, and crimes have destabilized individuals’ security in the short- and long-term
2. *Personal Freedom* – countrywide progress toward fundamental legal rights, individual liberties, and social tolerance
3. *Governance* – the extent to which there are checks and restraints on power and whether governments operate effectively and without corruption
4. *Social Capital* – the strength of personal and social relationships, social norms, and civic participation in a country
5. *Investment Environment* – the extent to which investments are adequately protected and are readily accessible
6. *Enterprise Conditions* – the degrees to which regulations enable businesses to start, compete, and expand
7. *Market Access and Infrastructure* – the quality of the infrastructure that enables trade and distortions in the market for goods and services
8. *Economic Quality* – how well a state’s economy is equipped to generate wealth sustainably and with the full engagement of its workforce
9. *Living Conditions* – the degree to which everyone experiences a reasonable quality of life, including material resources, shelter, essential services, and connectivity
10. *Health –* the extent to which people are healthy and have access to the necessary services to maintain good health, including health outcomes, robust systems, illness and risk factors, and mortality rates
11. *Education* – enrollment, outcomes, and quality across 4 stages of education (pre-primary, primary, secondary, and tertiary education) as well as the skills in the adult population
12. *Natural Environment* – the aspects of the physical environment that have a direct effect on people’s daily lives and changes that might affect future generations^11^

These pillars are fundamental elements for a prosperous country and enable a country to build up necessary structure to combat emergencies. Thus, why are these countries, at least in the beginning of the COVID-19 pandemic, highly influenced? One reason for the disparate COVID-19 impacts may be that these countries have better infrastructure, health care, and technical resources (eg, screening) to diagnose, follow up, report, and communicate.^12^ These advantages, particularly, more advanced and dynamic infrastructure, may, however, lead to the opposite outcome and accelerate the spread of the disease. On the other hand, less prosperous countries have neither advanced infrastructure to enable quick air/physical spreading, nor do they have well-organized health care systems to treat and report infected cases. People in these nations are initially isolated due to demography and geography. However, survivors need to access their needs by moving to more populated areas, movement which either reveals their diagnoses or exposes them to infection.^13,14^ Another explanation for the paradoxical association between COVID-19 outcomes and prosperity may be that other countries are systematically underreporting cases, have diverse political systems and agendas, older populations, or a population with specific underlying health conditions.^13–17^ For example, in some countries with Islamic or Buddhist culture, death certificates are issued with no autopsy, unless a crime is suspected. Consequently, the number of infected individuals in a pandemic is underreported, without any intention of subverting transparency.^18^ These reasons may partially explain why countries with advanced health care systems appear to be the most severely impacted by this pandemic, but the overall connection remains an outstanding question, which may change the course of management of future pandemics.

This study thus aims to clarify the association between prosperity and COVID-19 outcomes and its implications for future pandemic preparedness and management.

## METHODS

This is an observational study using information from 2 online registries. The number of infected individuals and deaths was obtained from worldometer.info, and the prosperity rank of each country was obtained from Legatum Institute’s Prosperity Index.^10,11^

### Obtaining the Number of Infected Persons and Deaths

The data from worldometer.info were collected on 4 separate occasions (May 21, May 28, June 4, and June 11, 2020). This site contains, among others, data on the actual number of infected cases and the number of deaths associated with COVID-19.^10^ Since population sizes vary significantly between countries, the number of total cases and deaths per million persons was used. Due to the large number of countries (n = 197) included in this registry, the first and the last 25 countries were initially included. However, many of the countries at the end of the COVID-19 ranking list did not have any prosperity ranking.^10^ Therefore, to include as many nations as possible, the 26 most infected countries on the list (1–26) and countries listed between 100 and 126 were included (n = 52) (Table 1).

**TABLE 1:** Included Countries 1–26 and 100–126 According to COVID-19 Ranking List During the Study Period and in 4 Different Occasions

	Ranked 21 May	Ranked 28 May	Ranked 4 June	Ranked 11 May
Countries 1–26	United States Russia Brazil Spain UK Italy France Germany Turkey Iran India Peru China Canada Saudi Arabia Chile Mexico Belgium Pakistan Netherlands Qatar Ecuador Belarus Sweden Switzerland Portugal	United States Brazil Russia Spain UK Italy France Germany India Turkey Iran Peru Canada Chile China Mexico Saudi Arabia Pakistan Belgium Qatar Netherlands Bangladesh Belarus Ecuador Sweden Singapore	United States Brazil Russia Spain UK Italy India Germany Peru Turkey Iran France Chile Mexico Canada Saudi Arabia Pakistan China Qatar Belgium Bangladesh Netherlands Belarus Ecuador Sweden South Africa	United States Brazil Russia UK Spain India Italy Peru Germany Iran Turkey France Chile Mexico Pakistan Saudi Arabia Canada China Bangladesh Qatar Belgium South Africa Belarus Netherlands Sweden Ecuador
Countries 101–126	Guinea-Bissau Hong Kong SriLanka Tunisia Latvia Lebanon Albania Mali Niger Cyprus Costa Rica Equatorial Guinea Zambia Paraguay Venezuela Burkina Faso Andorra Uruguay Georgia Jordan Haiti San Marino Malta Chad Sierra Leone Channels Islands	Slovenia Haiti Venezuela Guinea-Bissau Mali Lebanon Albania Hong Kong Tunisia Latvia Zambia Equatorial Guinea Nepal Costa Rica South Sudan Niger Cyprus Paraguay Burkina Faso Ethiopia Sierra Leone Uruguay Nicaragua CAR Georgia Jordan	Slovakia New Zealand Ethiopia Slovenia Mali Guinea-Bissau Equatorial Guinea Lebanon Albania CAR Costa Rica Nicaragua Hong Kong Zambia Tunisia Latvia Paraguay South Sudan Niger Cyprus Sierra Leone Madagascar Burkina Faso Andorra Uruguay Chad	Sri Lanka Iceland Lithuania Mali South Sudan Slovakia New Zealand Slovenia Nicaragua Costa Rica Guinea-Bissau Lebanon Albania Equatorial Guinea Mauritania Paraguay Zambia Madagascar Hong Kong Latvia Tunisia Sierra Leone Niger Cyprus Burkina Faso Jordan

### Prosperity Rank

A total number of 167 countries are ranked according to the sum of rankings for each of the 12 prosperity pillars listed in the Legatum Institute’s Prosperity Index list.^11^ The prosperity rank for each country on the COVID-19 ranking list was extracted from this index. Any country or territory mentioned in the COVID-19 ranking list that did not have a prosperity ranking was excluded.

### Other Indices

The World Economic Forum (WEF) global competitiveness index (eg, quality of infrastructure, education system), Word Bank country income classifications, and Transparency International’s Corruption Perception Index (to account for underreporting of cases/deaths in certain countries) were also used for potential association.

### Infection Fatality Rate

To simplify the data analysis, the infection fatality rate (IFR), that is, the number of deaths/number of infected cases, for each country on the list was calculated.^19^ The initial estimation of IFR by the WHO was 1.4%. However, this figure was revised to 2% on January 29 and February 10, 2020, and finally upward to 3.4% in March. This rate has varied between 0.5% and 14% in different countries and during various stages of the pandemic. The mortality rates are much higher than those of the seasonal flu.^20-26^

All the aforementioned information, that is, IFR, the COVID-19 ranking, and Prosperity Index score, was inserted in an Excel file and matched. A power analysis with a standardized statistical power of 0.80 and medium effect size of 0.5 premeditated the appropriate sample size to 200 observations. The observations were collected on 4 occasions to achieve the required number. Countries in the COVID-19 index that were not included in the Prosperity Index were excluded. This reduced the number of countries in the analysis to 49 on May 21; 52 on May 28; 51 on June 4; and 51on June 11. Once these data were compiled, an exploratory data analysis was performed.

### Statistics

The variables were defined in the computer program, Statistical Package for the Social Sciences 25.0 (SPSS; IBM Corp, Armonk, NY). The statistical significance was established at *P* < 0.05 and all tests were 2-tailed. The analysis stems primarily from descriptive data and regressions.

## RESULTS

**FIGURE 1:**
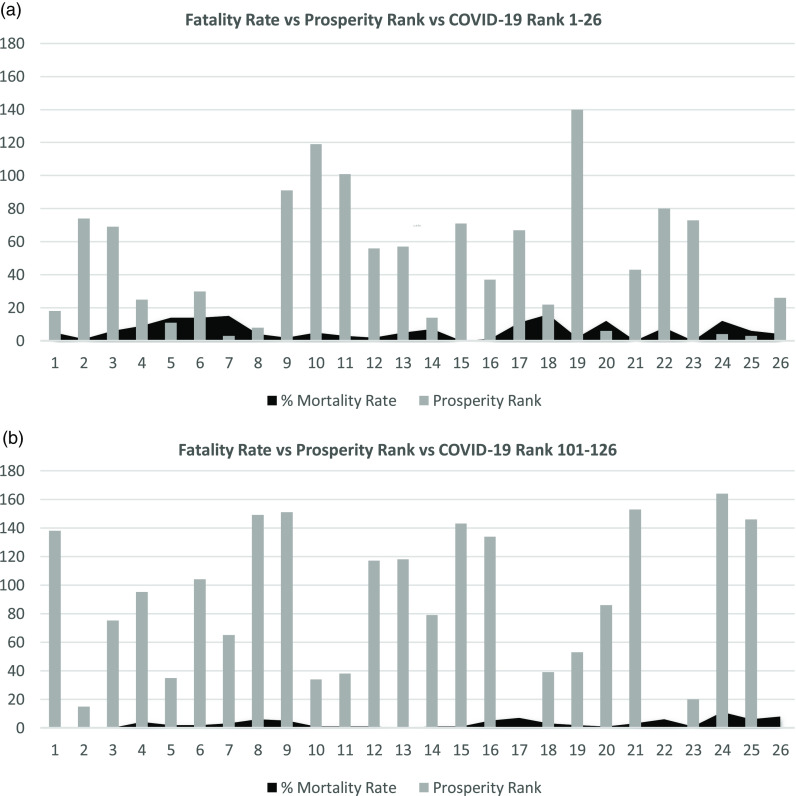
Showing the Correlation Between Fatality Rate and Prosperity in COVID-19 Ranked Countries on May 21, 2020.

**FIGURE 2:**
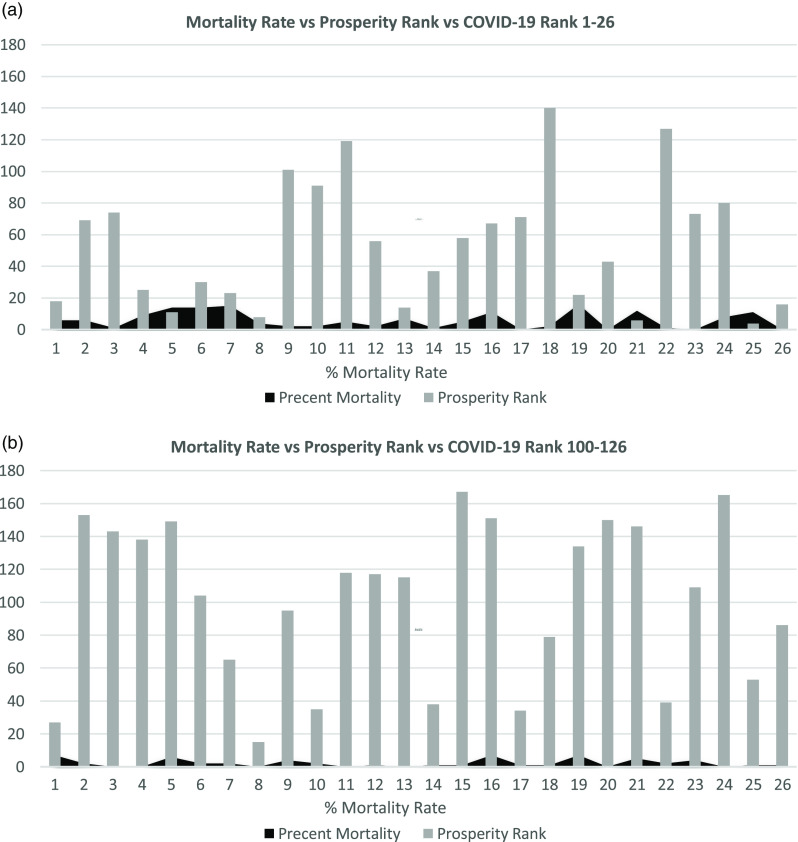
Showing the Correlation Between Fatality Rate and Prosperity in COVID-19 Ranked Countries on May 28, 2020.

**FIGURE 3:**
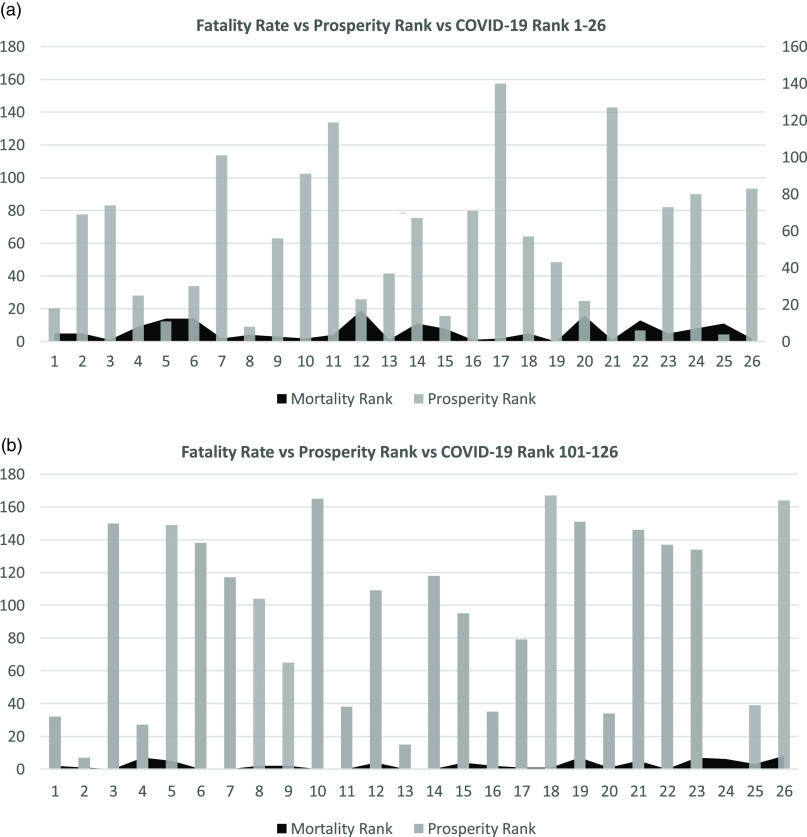
Showing the Correlation Between Fatality Rate and Prosperity in COVID-19 Ranked Countries on June 4, 2020.

**FIGURE 4:**
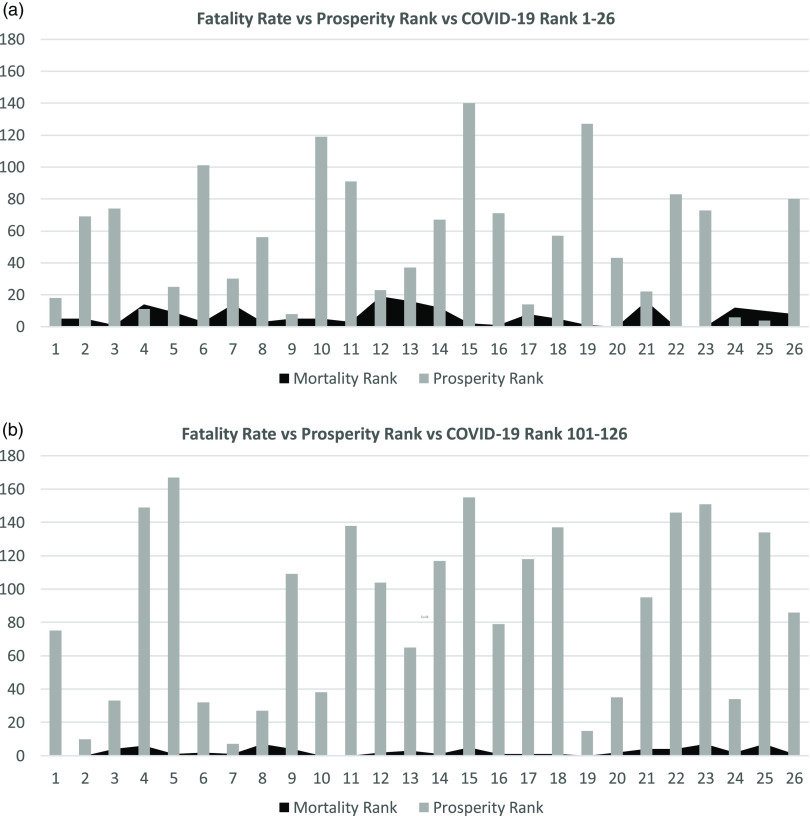
Showing the Correlation Between Fatality Rate and Prosperity in COVID-19 Ranked Countries on June 11, 2020.

Figures 1–4 show the relation between the fatality rate vs prosperity rank and COVID-19 rank. Each figure is presented as *a* and *b*. The former represents the data from the first 26 most significantly infected nations, and the latter shows the countries ranked 100–126 on the COVID-19 ranking list. The numbers on the horizontal axis represent the COVID-19 ranking of countries, with number 1 indicating the highest, and number 26 holding the lowest number of COVID-19 infections and deaths. In the prosperity-ranking list, low numbers represent countries with higher prosperity, whereas higher numbers are countries with lower prosperity. The position of each country is shown parallel to the y-axis (light bars). Smaller bars represent more prosperity, and vice versa. The dark curves represent IFR percentage, which is the risk of death for COVID-19 in each country. Taller bars mean a higher risk.

The figures demonstrate a combination of countries with high and low prosperity, indicating that COVID-19 has spread to all countries, irrespective of their prosperity level. However, the risk of being infected by the virus seems to be more extensive in prosperous countries. There are some exceptions: in Figure 1b, numbers 17, 22, and 26 represent countries/areas, Andorra, San Marino, and the Channel Islands, respectively, with no prosperity scale.

On May 28, the same pattern evolved, although a few countries changed places. However, most of the countries from the list on May 21 were there, showing higher risk for more prosperous nations to be infected by COVID-19 and consequently higher death rates (Figure 2a). In countries with lower rates of infection, both the prosperity and mortality chances were limited. The only exception was point 8, a prosperous Hong Kong with low mortality risk (Figure 2b) but a higher population.

Figure 3a and 3b shows the data from June 4 with the same pattern. Most of the countries remain on the list with few changes. In Figure 3b, Andorra is missing due to the lack of prosperity ranking. On June 11, there are still some changes in both lists of more and less prosperous countries. Some countries join the lists, whereas others move down but still show high mortality and morbidity (Figure 4a, 4b).

In order to follow up on the progress of the disease, the IFR from all 4 occasions were compared in all included (n = 29) countries. The changes in risk progress or regress during the period of study have no statistical significance. The IFR remains constant in most of the countries, despite changing positions. The increase of IFR in a few countries has been concordant with the sudden increase of infection or death in real time. The WEF global competitiveness index, Word Bank country income classifications, and Transparency International’s Corruption Perception Index were also matched against the COVID-ranking and Prosperity Index data. Still, they did not add any new information to the results.

The results of normality, measured by the Kolmogorov Smirnov test, did indicate total cases and deaths to be significantly skewed (Total Cases/1 M of population sig. 0.00, Deaths/1 M of population sig. 0.00). Due to the skewness and the fact that prosperity was presented as ranks, a non-parametric Spearman’s rho test was chosen to measure co-variations of the data sampled in May and June 2020. Extreme outliers were deleted from the test, that is, Qatar, Andorra, and San Marino. The test confirmed a significant correlation between prosperity, cases, and deaths at the 99% level (Tables 2 and 3).

**TABLE 2:** Ranks and Statistics of Prosperity, Total Cases Per 1 Million of Population

		Prosperity Rank	Total Cases of COVID-19 Per 1 M Population
**Prosperity** **Rank**	Correlation coefficient	1.0	-0.644
	Sig. (2-tailed)		0.00
**Total Cases of COVID-19 Per 1 M Population**	Correlation coefficient	-0.644	1.0
	**Sig. (2-tailed)**	**0.00**	

**TABLE 3:** Ranks and Statistics of Prosperity, Deaths Per 1 Million of Population (N = 201)

		Prosperity Rank	Total Deaths of COVID-19 Per 1 M Population
**Prosperity Rank**	Correlation coefficient	1.0	-0.622
	Sig. (2-tailed)		0.00
**Deaths from COVID-19 Per 1 M Population**	Correlation coefficient	-0.622	1.0
	**Sig. (2-tailed)**	**0.00**	

## DISCUSSION

This study indicates that a pandemic affects all nations irrespective of their prosperity, development, and the amount and type of measures they may implement after the outbreak, since measures and treatment are only useful when directed to the cause and in the early stages. As recently reported, because many cases of COVID-19 are asymptomatic and without any clinical signs, it is almost impossible to initiate any medical activity.^1-3,27^ Furthermore, there seems to be a correlation between COVID-19 infection and a nation’s grade of prosperity. Countries that are more prosperous seem to be more troubled, and the spread of the infection is faster. Consequently, the fatality rate also differs between countries and is higher in wealthy nations. The results of the non-parametric Spearman’s rho test, which measures co-variations of the data sampled in May and June 2020, confirmed this significant correlation between prosperity, infections, and the number of deaths at the 99% level (see Tables 2 and 3).

These are interesting observations that need to be explored in more detail and in larger samples since they may influence future planning in the management of pandemics. It is evident that similar unexpected pandemics will emerge in the future and traditional management strategies may be inadequate and health care resources insufficient.^20-28^ Consequently, there will be little chance, if any, to influence pandemics’ impacts and fatality rates during the incubation period. Therefore, a proactive approach should replace the reactive strategy in pandemics, as described in the WHO’s sustainable development goals.^29^ Such proactivity includes data-driven risk assessment, planning and mitigation strategies, and transparent and quick medical decision-making.

Although all elements in the Prosperity Index are crucial for the development of a country, they may create opportunity for emerging diseases, and higher risk for viral spread and mortality in a pandemic.^11^ Safety, security, and personal freedom enhance societal engagement, mass gatherings, traveling, and use of social media, and thereby attenuate physical distancing strategies, quarantine rules, and increased contact spreading, as well as rumors.^9,17,30-32^ Social capital enhances civic participation, changes norms and cultures, develops the needed social resiliency in responding to crisis, and enables flexible surge capacity. Its absence, on the other hand, results in sociocultural insufficiency and significant dependency on authorities and professional forces in the immediate response period to emergencies.^30,31^ The combination of a well-established investment environment, enterprise conditions, and market access and infrastructure promotes economic development and prosperity. It creates new mitigation and response opportunities and increases public access to services and basic necessities of life. However, if politicized, these factors influence medical and welfare decisions and result in unfair resource distribution. They can also force people in underserved communities to search for basic needs of survival and prevent both physical distancing and quarantine advantages.^15,30^

Poor living conditions and the natural environment help spread diseases and foster unhealthy individuals. Unhealthiness, in turn, increases the risk for adverse medical conditions and complications related to emergencies, including pandemics.^17,30,33^ Such conditions may also exist in a prosperous country. In a recent Swedish publication, large differences in excess mortality from March–May 2020 by country of birth in Sweden were reported when compared to the same period in 2019. The report indicated the shortcoming of the Swedish strategy for not accounting for difficulties of conducting voluntary physical distancing in neighborhoods with household overcrowding, dependence on public transport, and large proportions of service sector workers. Such neighborhoods are typically inhabited by a larger proportion of immigrants. The comparison of all-cause-mortality data by country of origin from 2016–2020 showed large disparities. The number of deaths among persons born in countries from which many refugees have migrated to Sweden in the last few decades was 220% higher from March–May 2020 compared with the mean between 2016 and 2019. In contrast, there was no increased mortality among persons ages 40–64 years and a 19% increase in the number of deaths of those ages over 65 years born in Sweden, the EU, or North America during these 3 months. These observations further illustrate the need for a dedicated and more diverse strategy in dealing with the pandemic.^17^

Educational initiatives can increase necessary public knowledge and skills to respond to crisis in adult and younger populations and enable the understanding and implementation of recommendations given by authorities. However, they must involve the right and appropriate target groups, be evaluated properly and continuously, and be repeated within a predesigned and evidence-based period.^30,31^ Finally, all of the aforementioned measures need a trustful and evidence-based governance to manage the crises, to increase the efficiency of measures, to ease up the implementation process, to prevent and mitigate the pandemic, and to enhance citizen’s social engagement. Defective governance results in concentrated power, economic corruption, limitation of democratic values, and missed mitigation and prevention opportunities.^15,34^

In prosperous countries, the risk for interaction between people and the spread of a pandemic seems to be more extensive. These nations have a well-developed infrastructure and logistics to support internal and international communications, as well as economic and enterprise activity. Shorter communication necessary between different parts of the country through the air and ground enables the disease to spread faster. This may explain the more rapid spread of COVID-19 and thus higher risk for infection and deaths in the current pandemic. However, although not to the same extent, less prosperous countries seem to catch up and suffer from the pandemic, since the shortcomings in public services and the lack of necessary life-items push isolated communities toward more intensive and higher density areas. These challenges may call for more up-to-date, sustainable infrastructure development and new public health strategies.^30,32,35-38^

Although this study may indicate that a pandemic is inevitable and hard to stop, it also encourages fundamental hygiene measures, including early and complete physical distancing strategies, knowledge of triggering factors, and ways that a pandemic can spread. These are crucial elements of public education and public health measures, which should be initiated long before any pandemic starts. The public health authorities should have the responsibility, mandate, and needed resources for initiating and implementing various activities to mitigate the risks for future pandemics proactively, to create more flexible surge capacity, and promote a multidisciplinary workforce, structure, and system.^30,31,35-37^

No country is immune to the consequences of a pandemic. Each nation has its own unique resources, knowledge, competences, and capabilities. For example, in Brazil, Pakistan, and Ethiopia, staff between the ages of 18 and 30, with an anticipated low risk of infection, were recruited to support the older population, who were isolated at their homes. The strategy resulted in positive outcomes and was recommended for further implementation in other countries.^30,35,36^ Nevertheless, various nations have to consider several political, economic, sociocultural, and religious factors, of which the most decisive might be the political and economic factors, which directly influence sociocultural and spiritual factors.^3^ Individual countries remain responsible for the management of a crisis within their borders and should continue developing relationships with leading health care organizations, such as the WHO. Global health institutions, such as the WHO and International Federation of Red Cross and Red Crescent Societies, should be free of political and economic influence.^15,36,39,40^ Public health should strengthen its position as the leader in crisis by activating civilians and health care resources with guidelines and recommendations adjusted to each country.^3,30^ This is necessary to develop early warning systems and educational initiatives adjusted to different types of crises. A pandemic warning system instructs the population to act and implement various measures in different phases of a pandemic.^41^ Targeted educational initiatives will empower civilians and enhance interagency collaborations.^30,31^

### Limitations

The main limitation of this study is the changing variables in the COVD-19 ranking site, including the number of deaths, infected population, the population density, and reporting validity. Additionally, in this study, 1 factor (ie, prosperity) was studied, whereas several other factors, such as preparedness, risk awareness, resilience, and adaptability, also need to be explored. Furthermore, the data for each country on the Prosperity Index were compared with data from the Corruption Perception Index, and no added information could be obtained. However, no statistical analysis was performed to verify the significance of the results. Finally, there is no comparable study to match with the results of this study.

## CONCLUSION

There have been pandemics before, and new ones will occur. The threat of several additional waves of COVID-19 seems imminent if mitigation efforts fail and a vaccine is not found soon. New emerging pandemics may have no name, no symptoms, no treatment, and no vaccines.^27^ In order to increase the likelihood of successfully managing future events, it is important to consider preexisting health security, a valid PBM organization, medical decision-making, communication, continuous assessment, triage, treatment, early and complete physical distancing strategies, and logistics.^15,17,30^ These elements cannot be taught on-site and on occasion.

This paper aimed to be thought-provoking and debatable to emphasize the need for innovative and regular educational activities for all stakeholders committed to safeguarding our future defense systems concerning diagnostic, protection, treatment, and rehabilitation in pandemics as well as other emergencies. Nations, irrespective of prosperity, are neither immune nor able to handle a pandemic alone. New initiatives should be undertaken to empower all countries, and future studies of prosperity will help identify new global strategies to effectively manage future pandemics.
